# The effect of mentoplate application on the condyle

**DOI:** 10.1186/s12903-024-04506-0

**Published:** 2024-06-24

**Authors:** Gorkem Tekin, Ayşe Tugce Ozturk Kocak, Batuhan Kuleli, Taner Ozturk, Omur Dereci, Nesrin Saruhan Kose, Mehmet Ugurlu, Yasin Caglar Kosar, Gizem Caliskan

**Affiliations:** 1 Faculty of Dentistry, Department of Oral and Maxillofacial Surgery, Osmangazi University, Eskisehir, Turkey; 2 Specialist in Oral and Maxillofacial Radiology, Private Practice, Antalya, Turkey; 3 Faculty of Dentistry, Department of Orthodontics, Osmangazi University, Eskisehir, Turkey; 4 Faculty of Dentistry, Department of Orthodontics, Kayseri, Turkey

**Keywords:** Angle Class III, Fractal, Mandibular condyle

## Abstract

**Background:**

The aim of the study was to investigate the changes occurring in the mandibular condyle by using mentoplate together with rapid maxillary expansion (MP-RME) treatment in the correction of skeletal class III relationship, using fractal analysis (FA).

**Methods:**

The sample consisted of 30 individuals (8–11 years) diagnosed with skeletal Class III malocclusion who underwent MP-RME treatment. Archival records provided cone-beam computed tomography (CBCT) images taken at two intervals: before MP-RME treatment (T0) and after treatment (T1). The CBCT images were obtained using standardized settings to ensure consistency in image quality and resolution. The trabecular structures in the bilateral condyles at both T0 and T1 were analyzed using FA. The FA was performed on these condylar images using the Image J software. The region of interest (ROI) was carefully selected in the condyle to avoid overlapping with cortical bone, and the box-counting method was employed to calculate the fractal dimension (FD). Statistical analysis was conducted to compare the FD values between T0 and T1 and to evaluate gender differences. The statistical significance was determined using paired t-tests for intra-group comparisons and independent t-tests for inter-group comparisons, with a significance level set at *p* < 0.05.

**Results:**

The analysis revealed no statistically significant differences in the trabecular structures of the condyles between T0 and T1 (*p* > 0.05). However, a significant gender difference was observed in FA values, with males exhibiting higher FA values in the left condyle compared to females at both T0 and T1 (*p* < 0.05). Specifically, the FA values in the left condyle increased from a mean of 1.09 ± 0.09 at T0 to 1.13 ± 0.08 at T1 in males, whereas in females, the FA values remained relatively stable with a mean of 1 ± 0.09 at T0 and 1.03 ± 0.11 at T1.

**Conclusion:**

The findings indicate that MP-RME therapy does not induce significant alterations in the trabecular structure of the mandibular condyle. These results suggest the treatment’s safety concerning the structural integrity of the condyle, although the observed gender differences in FA values warrant further investigation.

## Backround

Skeletal Class III malocclusion encompasses a diverse group of dentofacial anomalies, primarily defined by the forward positioning of the mandible relative to the upper maxilla across its various phenotypic sub-clusters [1]. True Class III malocclusion often has a positive family history. Cephalometric findings typically include an increased SNB angle, a small or decreased SNA angle, retroclination of the lower incisors, and a lower incisor mandibular plane angle [2]. Treatment of jaw deformities requires a multidisciplinary approach, and its treatment is a very complex and challenging task for clinicians [3, 4].

There are various treatment options available for the management of Class III malocclusion [2]. Early interventions performed at a young age are crucial to prevent the worsening of Class III malocclusions and eliminate the need for surgery [5]. The most suitable time for orthopedic intervention is typically during the pre-adolescent or adolescent period [6].

Maxillary protraction for the orthopedic treatment of Class III malocclusions resulting from maxillary deficiency can be achieved using various appliances. Traditionally, maxillary protraction is accomplished through extraoral traction using various types of facemasks [7]. Research findings indicate that the combined treatment involving RME and a facemask leads to advancements in the maxilla, a clockwise rotation of the mandibular plane, a counterclockwise rotation of the palatal plane, forward movement of maxillary incisors, backward movement of mandibular incisors, and elevation of maxillary molars [8]. Additionally, it has been shown that facemask treatment also leads to morphological adaptation of the temporomandibular joint and displacement of the mandibular condyle [9]. Studies have reported that orthodontic procedures can induce mechanical stress on the condyle, which may initiate condylar resorption or accelerate pre-existing resorption [10]. Alternatively, the utilization of skeletal anchorage in the mandible obviates the necessity for supplementary extraoral devices, potentially enhancing patient acceptance and compliance. In a particular study group, MP is one of the anchorage devices that is used for maxillary protraction. It is positioned subapically beneath the lower incisors and can be introduced prior to the emergence of the canines [11].

One of the methods utilized in dentistry to identify changes in alveolar bone is FA [12]. FA, calculated from radiographic data, serves as a valuable tool for interpreting the microstructural characteristics of trabecular bone [13]. FA, despite its limitations, represents a straightforward and non-invasive method that yields objective outcomes, unaffected by variables such as projection geometry or radiation dosage [14]. Higher FA values represent a complex bone structure characterized by increased density and fewer pores within the trabecular architecture [15]. Visual assessment of trabecular changes in the bone components of Temporomandibular Joint (TMJ) may not always be feasible, but overcoming this obstacle can be achieved through FA measurements [13].

It is possible to encounter some difficulties in radiographic methods used to evaluate bone changes in the TMJ. Obtaining a clear and precise image of the area is important, but may be difficult due to the overlap of adjacent structures, the condyle being at different angles, limited mouth opening in some patients, and the presence of artifacts [16]. Despite these difficulties, radiographic imaging is still an important tool in evaluating changes in bone structure. To overcome these difficulties, combining radiographs taken from different angles and with different techniques or using other may also be considered [17]. Computed Tomography (CT)is a frequently used 3D imaging technique that creates detailed images of internal structures that aid diagnosis and facilitate treatment. CT imaging provides valuable information about the cortical and internal structure of bone in three planes [18]. According to Honey et al. [19]. , the radiation dose exposed is lower in CBCT than in CT, and bony changes in the TMJ can be visualized better with CBCT. In addition, CBCT provides 3D image quality of the highly mineralized tissue in the maxillofacial region with minimal distortion, and the cost is also low [20].

It is not clear how the results of MP applied in the orthopedic treatment of early class III malocclusion affect the skeletal stability of the trabecular structure. With this study, the positive or negative effects of the success of the results in clinical practice on TMJ will be determined. The studies investigating the effects of MP-RME treatment on the dentoskeletal complex are limited, and there is no study evaluating its effects on the mandibular condyle. Therefore, we aimed to assess the impact of MP-RME treatment on the trabecular structure of the condyle. The null hypothesis of this study was that the combined treatment of MP-RME affects on the trabecular structure of the mandibular condyle.

## Materials and methods

### Sample

This retrospective study included 30 patients (14 males and 16 females) with ages ranging from 8 to 11 years (mean:9.93 ± 0.78). Ethical approval was obtained from the Eskisehir Osmangazi University Non-Interventional Clinical Research Ethics Committee with approval number 2023/41, and the study was organized with conjunction to the ethical principles of the Helsinki Declaration for medical research involving human subjects. All participants were provided with verbal and written information about the purpose and methodology of the study, and signed informed consent forms were obtained.

The inclusion criteria for the study were as follows:

- Patients with Class III molar relationship and anterior crossbite or end-to-end occlusion in late mixed or early permanent dentition.
- ANB < 0, SNA < 80, Wits < -2 mm.
- Cervical vertebral maturation stages ranging from CVMS I to III.
- Patients without congenital anomalies, upper airway problems, or a history of previous orthodontic treatment.

Exclusion criteria involved insufficient demographic and radiographic data, those who did not attend routine follow-ups, individuals with poor oral hygiene, craniofacial anomalies, a history of trauma to the TMJ region, TMJ disorders, missing teeth, systemic diseases affecting bone metabolism, or those taking medications that could affect bone metabolism. MR and CBCT images were used to diagnose TMJ disorder.

### Treatment protocol

**Fig. 1:**
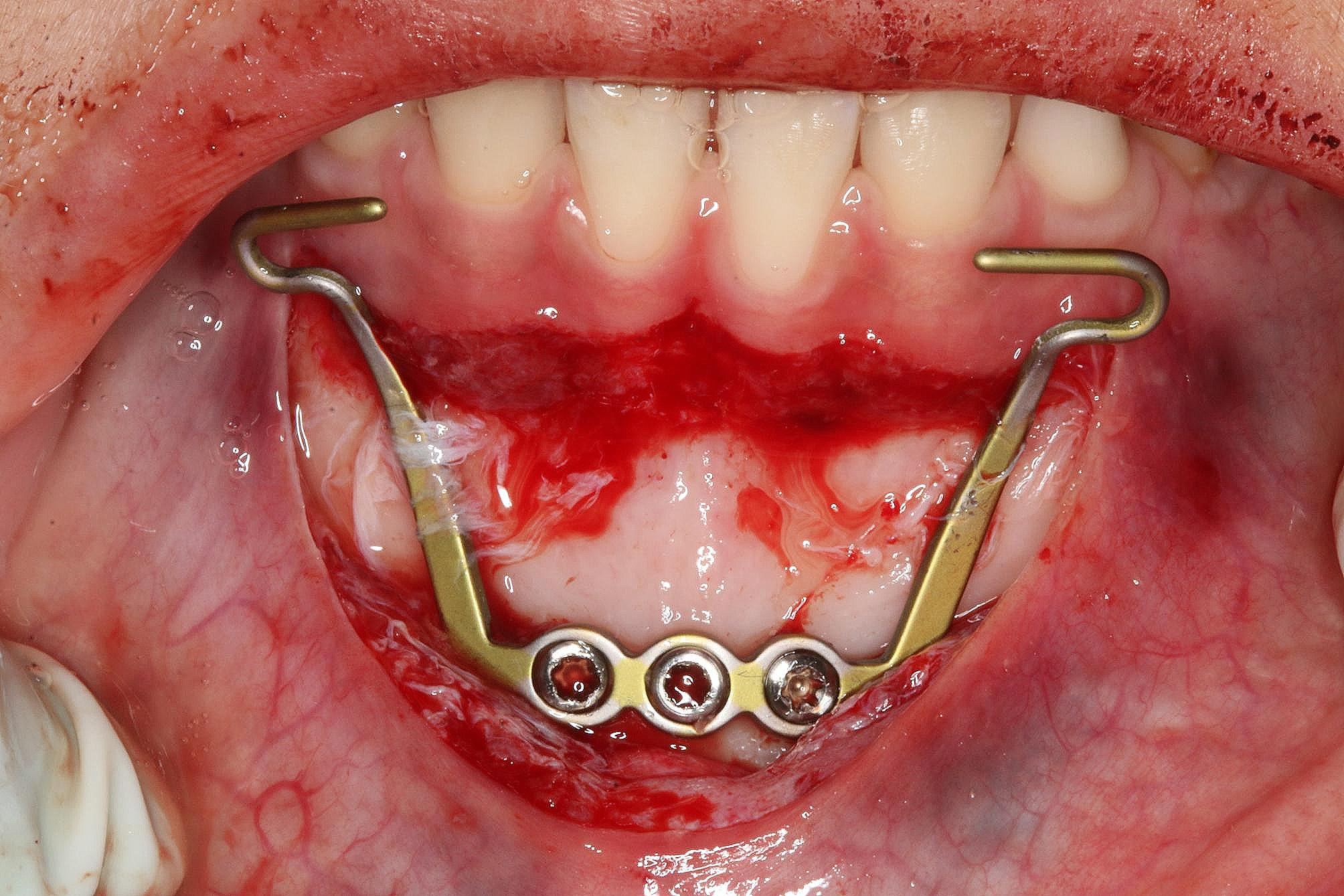
Surgical operation for MP placement

MP applications were performed under local anesthesia in the operating room of the Department of Oral and Maxillofacial Surgery at Eskisehir Osmangazi University Faculty of Dentistry. The used MP was described as consisting of a titanium plate with a width of 18 mm, no bar, and three holes in the 30 mm extensions (PSM Medical Solutions, Tuttlingen, Germany). A horizontal incision within the boundaries of the attached gingiva on the vestibular surface of the lower incisors was made, and a mucoperiosteal flap was elevated. After ensuring the plate’s compatibility with the anatomical region and bending the transmucosal extensions to the appropriate height mesially for the attachment of elastics, the titanium plate was fixed to the bone surface using 2.0 × 5 or 2.0 × 7 mm self-drilling mini screws (TX bone screw, PSM Medical Solutions, Tuttlingen, Germany) (Fig. 1), and the flap was closed. A waiting period of 7–10 days was observed for soft tissue healing. A force of 250–400 g was applied on one side with intermaxillary elastics applied between the hooks on the molar bands in the upper jaw and the hooks of the MP in the lower jaw seen inside the mouth.

### Study design

Before (T0) and after (T1) MP-RME treatment, CBCT images of the patients were obtained. Trabecular changes in the right and left mandibular condyles were analyzed using FA. The FA measurements were performed by an experienced radiologist. The analyses were conducted on CBCT images obtained both before and after treatment. The CBCT images used in the study were acquired using the Planmeca Promax 3D series Mid type CBCT device (Planmeca Promax 3D Mid Proface, Helsinki, Finland). The imaging parameters were as follows: tube voltage = 94 kVp, X-ray tube current = 14 mA, rotation = 360°, scanning time = 27 s, and voxel size = 0.600 μm with a slice thickness of 0.3 mm.

### Fractal analysis

**Fig. 2:**
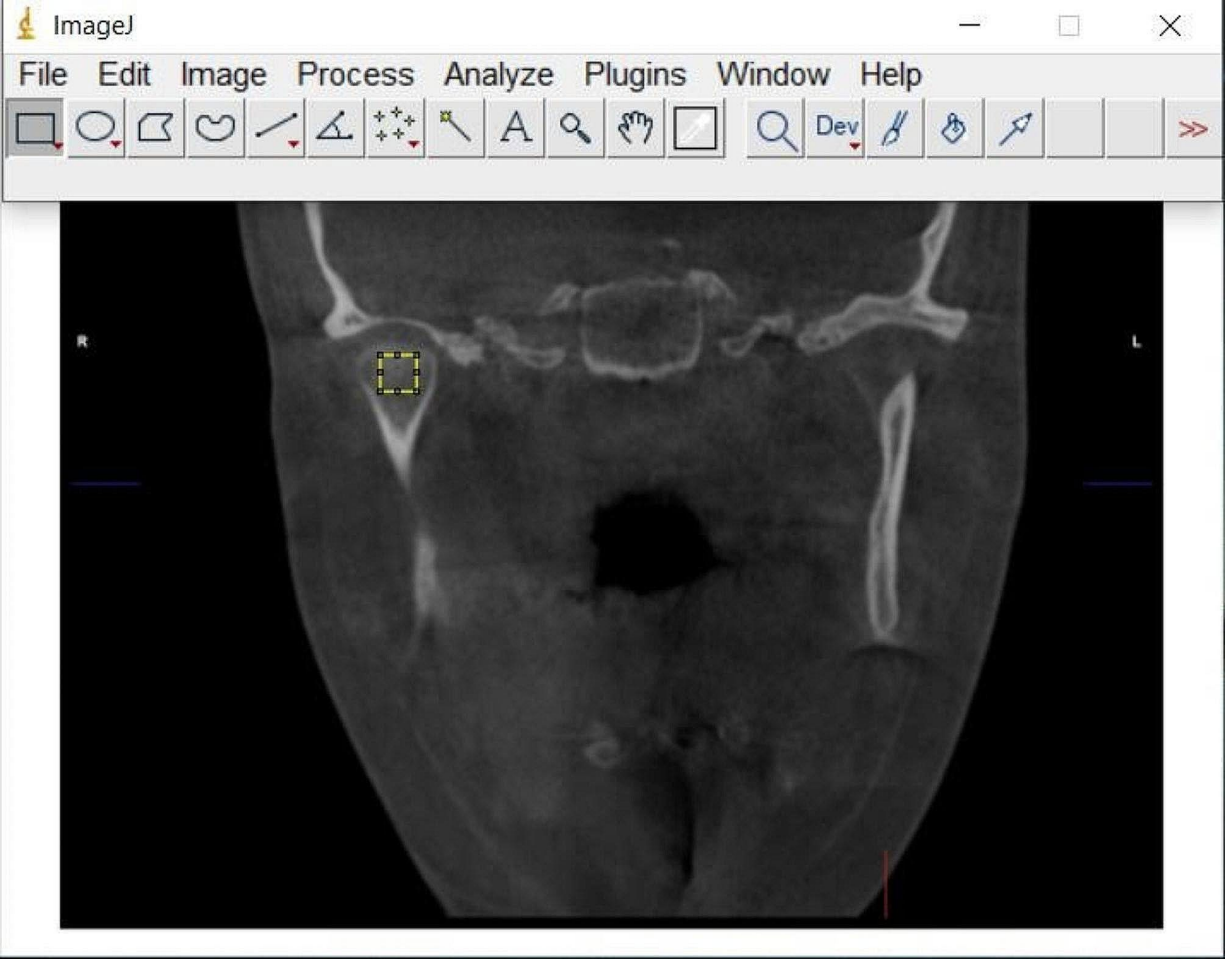
ROI selection on coronal CBCT image

**Fig. 3:**
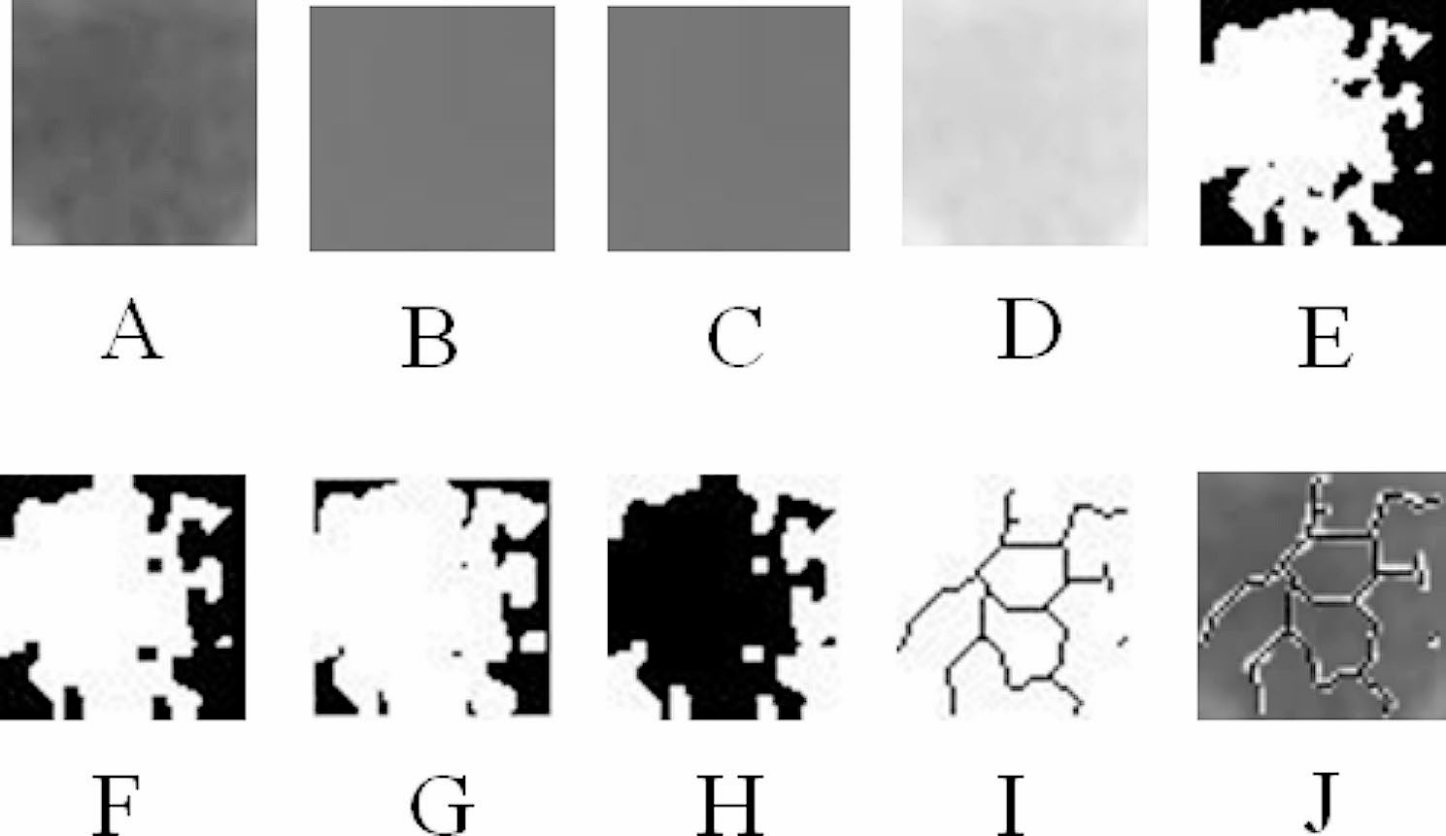
Image processing for FA. (a) Duplicated ROI, (b) Blurred image, (c) Subtracted image, (d) Added image 128 Gy values (e) Thresholded image (f) Eroded image, (g) Dilated image, (h) Inverted image, (i) Skeletonized image, (j) Overlay image of the original image and the analyzed skeleton

CBCT images were examined on 0.3 mm thick sections. Similar to previous study, coronal images passing through the center of the condylar head were selected [21]. These images were saved as jpeg. Two images were taken separately for the right and left side of mandibular condyles. Image J, a java image processing and analysis program, was used for FA. The program was downloaded for free from https://imagej.nih.gov. The method of White and Rudolph’s was used to prepare images for FA [22]. In the coronal image, a 25 × 25 pixel square ROI (Region of interest) was selected from the condyle head (Fig. 2). ROIs were selected from trabecular bone regions, avoiding cortical bone. The selected ROI was duplicated (Fig. 3a). The image was blurred by selecting the Gaussian blur filter (Fig. 3b). The blurred image was removed from the original image to eliminate noise (Fig. 3c). 128 Gy values were added at each pixel location (Fig. 3d). The threshold option was used to convert the image to black and white (Fig. 3e). The image was eroded, then the pattern was made more prominent with the dilate option (Fig. 3f and g). The image was prepared for FA by applying the invert and skeletonize options (Fig. 3h, i and j). The complexity of the trabecular structure was measured using the fractal box counting method.

### Statistical analysis

The analyses were conducted using SPSS 26 software. Continuous data were tested for normality using the Kolmogorov-Smirnov and Shapiro-Wilk normality tests. Since the data showed a normal distribution, the analyses were performed using Independent Samples-t test, Paired Samples-t test and Wilcoxon signed-rank test. The relationship between treatment duration and other parameters was examined with Pearson Correlation analysis. The average of the FA change occurring in the right and left mandibular condyle was taken as the TMJ - FA change value, and a linear regression model was created to determine the factors affecting the change. A p-value of less than 0.05 was considered statistically significant.

### Method error

All images were evaluated by the same observer. The images of ten randomly selected patients were re-evaluated three weeks later to calculate intra-observer reliability. Intraclass Correlation Coefficients (ICC) were calculated to determine the reliability between measurements. Cronbach alpha values for the first and second measurements were found between 0.786 and 0.953. In this way, the reliability of the measurements was confirmed.

## Results

While the SNA (*p* < 0.001) and ANB (*p* < 0.001) angles significantly increased thanks to the orthopedic force applied through the MP, the maxilla was skeletally protracted without any movement in the mandible, since there was no significant change in SNB (*p* = 0.258, Table 1). Additionally, wits appraisal measurement also showed a positive improvement (*p* < 0.001, Table 1). There is a significant difference between T0 (*p* = 0.012) and T1 (*p* = 0.008) left condylar FA measurements in both male and female individuals (Table 1). In female individuals, the mean left condylar FA at T0 and T1 is significantly lower compared to males (For males, the mean left condylar FA at T0 = 1.09, while for females, the mean left condylar FA at T0 = 1.00). Pre-treatment and post-treatment right TMJ measurements do not show a significant difference based on gender. When considering the entire patient group, there is no significant difference in FA measurements between T0 and T1 in the condyle (*p* = 0.765, Table 1).

**Table 1:** Comparison of Condyle FA and cephalometric variables before and after treatment

	T0		T1		*P* values
	Mean	SD	Mean	SD	
**Left TMJ (Female)**	1.00	0.09	1.03	0.11	0.418*
**Left TMJ (Male)**	1.09	0.09	1.13	0.08	0.153*
**P values**	0.012***		0.008***		
**Right TMJ**	1.03	0.12	1.03	0.10	0.765*
**Total TMJ**	1.04	0.11	1.05	0.11	0.360*
**SNA**	79.29	2.27	81.76	2.00	< 0.001*
**SNB**	81.26	2.39	81.18	2.45	0.258**
**ANB**	-1.90	2.14	0.59	1.75	< 0.001*
**Wits Appraisal**	-4.01	1.82	-1.58	1.19	< 0.001**

SD: Standard Deviation. * Paired samples-t test. ** Wilcoxon signed-rank test

*** Independent samples-t test

The correlation of treatment duration and FA change in TMJ with the change in other parameters is given in Table 2. It was determined that there was a significant positive correlation between treatment duration and SNA (*p* < 0.001), ANB (*p* = 0.004) and Wits appraisal (*p* = 0.020) parameters. However, no significant correlation was found between treatment duration and TMJ - FA change. When the factors affecting the TMJ - FA change of the condyles were examined with a linear regression model (Table 3), it was determined that gender (*p* = 0.024) and the interaction of gender and duration (*p* = 0.010) of use had a significant effect.

**Table 2:** Treatment Duration and TMJ – FA change correlation matrix

Variables	Treatment Duration		TMJ – FA Change	
	Pearson’s *r* value	*P* value	Pearson’s *r* value	*P* value
TMJ - FA Change	-0.012	0.644		
Age	-0.310	0.098	-0.100	0.584
SNA difference	0.580***	< 0.001	-0.060	0.754
SNB difference	0.090	0.634	-0.220	0.242
ANB difference	0.0510**	0.004	-0.050	0.793
Wits appraisal difference	0.420*	0.020	-0.080	0.693

TMJ: Temporomandibular joint. FA: Fractal Analysis. *p < 0.05. **p < 0.01. ***p < 0.001.

**Table 3:** Mean TMJ – FA change linear regression results

Predictor	Estimate	SE	95% CI		t	*p*
			Lower	Upper		
Intercept ^a^	1.59	2.16	-2.93	6.12	0.74	0.470
SNA difference	0.07	0.05	-0.03	0.17	1.38	0.185
SNB difference	-0.05	0.05	-0.15	0.04	-1.22	0.238
ANB difference	-0.06	0.04	-0.15	0.02	-1.51	0.147
Wits appraisal difference	-0.02	0.02	-0.06	0.02	-0.95	0.353
Gender:						
Female – Male	-1.74	0.71	-3.23	-0.26	-2.45	***0.024***
Treatment Duration	-0.06	0.14	-0.36	0.23	-0.43	0.673
Age	-0.11	0.21	-0.56	0.33	-0.53	0.603
Age ✻ Treatment Duration	0.00	0.01	-0.03	0.03	0.24	0.813
Gender ✻ Treatment Duration:						
Treatment duration ✻ (Female – Male)	0.06	0.02	0.02	0.10	2.84	***0.010***
Age ✻ Gender:						
Age ✻ (Female – Male)	0.08	0.05	-0.03	0.20	1.56	0.134

^a^ Represents reference level. R^2^: 0.42. Overall model test p value: 0.262. Gender reference level was given female.

Note: The average of the FA change occurring in the right and left TMJ was taken as the mean TMJ - FA change value.

## Discussion

There are various opinions regarding the timing of treatment for Class III malocclusions and the effects of treatments in different age groups. Researchers advocating for early intervention have asserted that orthopedic effectiveness increases with treatment initiated in the early developmental stages, emphasizing that the outcomes primarily manifest at the skeletal level [6]. Another treatment approach is to wait for the completion of growth and subsequently implement either dental camouflage treatment or orthognathic surgery [23]. In our study, patients whose growth and development were not yet completed, ranging in age from 8 to 11, were included to assess orthopedic effects.

The treatment of Class III malocclusions varies depending on the origin (maxillary retrognathism, mandibular prognathism, or a combination of both), etiology, severity, and the individual’s stage of growth and development. Current treatment methods range from functional appliances to extraoral appliances such as facemasks and chin caps [24]. It has been reported that the force exerted by the chin-cup in a posterosuperior direction is directly applied to the mandibular condyle, which may lead to internal irregularities in the TMJ [25]. Wendi et al. [24] analyzed 61 patients with Class III malocclusion in their study, treating them with chin cap and facemask. According to the treatment outcomes, both chin cap and facemask applications yielded similar positive results. They reported that early chin cap treatment did not have adverse effects on the temporomandibular joints, but despite these findings, they emphasized the need to discuss the effectiveness of chin cap treatment, especially in terms of the risk of harm to the condyles. Wilmes et al. [11] reported that the application of Hybrid Hyrax and MP has several advantages over other appliances, such as the direct application of force to skeletal structures, the absence of the need for extraoral appliances, the stability and safe use of anchorage, and the ease and cost-effectiveness of the placement process. Katyal et al. [26]. treated 14 patients (7 males and 7 females), aged between 7.8 and 12.9 years (mean age 10.4 ± 1.7), using the hybrid Hyrax-MP combination. The authors evaluated the changes before and after the treatment using a lateral cephalogram. The mean sagittal changes revealed an increase in the SNA° by 2.1 ± 2° (*p* = 0.002), ANB° by 1.9 ± 1.8° (*p* = 0.002), and Wits appraisal by 3.4 ± 2.7 mm (*p* < 0.001). Additionally, it was observed that there was an overjet reduction of 2.0 ± 2.2 mm (*p* = 0.005). In our study, we evaluated patients treated with MP-RME, a combination that is rare in the literature but highly valuable for research purposes. Similar to Katyal et al., our study found statistically significant increases in SNA (2.47°), ANB (2.49°), and Wits appraisal (2.43 mm) values. We believe that our study results are consistent with the literature and demonstrate the successful application of this treatment approach.

FA can be employed to numerically determine and reveal the shape and size of structures [27]. In dentistry literature, there have been numerous studies that utilized FA analysis to evaluate bone structure [28, 29]. TMJ is utilized to examine systemic diseases affecting it, bone healing around implants, and the impact of periodontal disease on bone microstructure [13]. In the field of orthodontics, it is employed to examine the activity of the midpalate suture, assess the impact of orthodontic treatment duration on the mandibular bone, evaluate bone microstructure, and investigate the bone surrounding impacted maxillary canines [30, 31]. Furthermore, It is widely accepted within the academic community that the FA assessed through radiographic analysis is a reliable indicator of variations in trabecular bone density and mineral loss [22, 32]. The anisotropic nature of trabecular bone and variations in trabecular thickness are believed to yield diverse outcomes in studies focusing on FA [33]. Due to its previously demonstrated validity, in this study, we utilized FA to assess potential structural changes in the mandibular condyle following the use of MP-RME. In this study, among the various methods available for calculating FA, the most widely adopted approach, which is box counting, was employed. The box counting method is commonly used to estimate the fractal dimension of a fractal, and it can work even with low-resolution images. This method involves the enumeration of boxes containing trabeculae, computation of the box sizes, and subsequent plotting of the data on a logarithmic scale graph to ascertain the FA.

Ertuğrul [34] investigated the bone density in the mandibular condyle before and after treatment among different malocclusion groups. Author reported that the bone density in the condyles of groups treated with facemask and chin cap decreased according to FA results, and this decrease was statistically significant (*p* < 0.05) Author suggested that the reason for this decrease was the impact of the orthodontic treatment on the condylar region. However, author mentioned the need for further clinical studies to confirm this hypothesis [34]. Ertuğrul et al. [35]. , another study, compared transparent aligners with conventional braces and examined their effects on the condyle. The authors reported a decrease in condylar fractal values after treatment in patients undergoing orthodontic treatment with clear aligners. They attributed this reduction to the impact of clear aligners on the condylar region, suggesting that clear aligners could affect the temporomandibular joint, consequently influencing the condyle [35]. Gümüş et al. [36]. , functional appliances’ effects on the mandibular condyle in their study and found no significant difference in FA values of the condylar region before and after treatment. According to the results of this study, the trabecular structures of the condyles appear to be similar before and after treatment. Gulec et al. [12]. used the FA method to evaluate trabeculation in the condylar region in patients diagnosed with bruxism and found low FA values at the head of the right mandibular condyle to be statistically significant. Although the authors reported that low FA values may be related to absorptive variations caused by occlusal forces due to bruxism, significant results were observed only in the right condyle region, and the authors suggested that this difference may be due to unilateral chewing habits. Studies have shown decreased bone density in the mandibular condyle following treatments with facemasks and chin caps, suggesting potential negative impacts on the condyle. In addition, it has been found significant according to the literature that forces acting on the condyle, such as bruxism, reduce trabeculation in the condyle region. However, our study, focusing on the effects of MP-RME on the mandibular condyle, found no statistically significant changes in fractal analysis (FA) values pre- and post-treatment, indicating that this method does not adversely affect the condylar bone density.

Arsan et al. [15]. found that individuals with temporomandibular joint disorders, including disc displacement with reduction or without reduction, exhibited lower FA values in the mandibular condyles compared to the control group. The study identified a decrease in FA values in the condylar region when erosive and sclerotic changes were present. Cesur et al. [28]. conducted an evaluation of the impacts of functional appliance treatment on the mandibular trabecular structure using FA. Their study reports that when comparing pre- and post-treatment FA values between genders within both study groups, notable differences emerged. Specifically, it was observed that in the treatment group, pre-treatment FA values were significantly higher for girls in the right condylar process, while in the control group, higher FA values were recorded for boys in the left condylar process. Furthermore, the authors found no significant disparity in post-treatment FA values between girls and boys. When considering the entire patient group, there is no significant difference in FA measurements at T0 and T1 for the condyle. In other words, there is no substantial change observed in FA measurements between the initial assessment (T0) and the follow-up assessment (T1) for the condyle when looking at the entire patient cohort. While our study found gender differences in FA values, the overall changes before and after treatment were not significant, suggesting that MP-RME is a safe option for early intervention without detrimental effects on the condylar region.

### Limitation

The study’s duration of follow-up (from before treatment to after treatment) may be relatively short to capture long-term changes in trabecular structures. While FA is a valuable tool, the choice of the box counting method might have its own limitations. Alternative FA techniques could be explored for comparison. In addition, the small sample size is also a limitation of the study.

## Conclusion

The combination of MP-RME treatment did not show a notable impact on the trabecular structure of the mandibular condyle in patients with Class III malocclusion. Consistency in pre- and post-treatment condylar FA measurements was observed within both male and female groups, but a gender-based comparison revealed higher measurements in males. Given the relatively stable FA values of the condyles after treatment, it can be argued that the application of MP-RME did not adversely affect the mandibular condyles. The study’s null hypothesis was refuted.

## Data Availability

The datasets used and/or analysed during the current study are available from the corresponding author on reasonable request.

## Abbreviations

CBCT: Cone-beam computed tomography
FA: Fractal analysis
MP: Mentoplate
RME: Rapid maxillary expansion
ROI: Region of interest
CVMS: Cervical vertabra maturation stage
TMJ: Temporomandibular joint
